# Identification of glycogene signature as a tool to predict the clinical outcome and immunotherapy response in breast cancer

**DOI:** 10.3389/fonc.2022.854284

**Published:** 2022-09-14

**Authors:** Shuai Lin, Zengqi Tan, Hanxiao Cui, Qilong Ma, Xuyan Zhao, Jianhua Wu, Luyao Dai, Huafeng Kang, Feng Guan, Zhijun Dai

**Affiliations:** ^1^ Department of Oncology, The Second Affiliated Hospital of Xi’an Jiaotong University, Xi’an, China; ^2^ Key Laboratory of Resource Biology and Biotechnology in Western China, Ministry of Education, Joint International Research Laboratory of Glycobiology and Medicinal Chemistry, College of Life Sciences, Northwest University, Xi’an, China; ^3^ Department of Breast Surgery, The First Affiliated Hospital, College of Medicine, Zhejiang University, Hangzhou, China

**Keywords:** glycogene, MGAT5, breast cancer, prognosis, immune microenvironment

## Abstract

**Background:**

Breast cancer is one of the most important diseases in women around the world. Glycosylation modification correlates with carcinogenesis and roles of glycogenes in the clinical outcome and immune microenvironment of breast cancer are unclear.

**Methods:**

A total of 1297 breast cancer and normal cases in the TCGA and GTEx databases were enrolled and the transcriptional and survival information were extracted to identify prognostic glycogenes using Univariate Cox, LASSO regression, Multivariate Cox analyses and Kaplan-Meier method. The immune infiltration pattern was explored by the single sample gene set enrichment method. The HLA and immune checkpoint genes expression were also compared in different risk groups. The expressions of a glycogene MGAT5 as well as its products were validated by immunohistochemistry and western blotting in breast cancer tissues and cells.

**Results:**

A 19-glycogene signature was identified to separate breast cancer patients into high- and low-risk groups with distinct overall survival rates (*P* < 0.001). Compared with the high-risk group, proportion of naive B cells, plasma cells and CD8^+^ T cells increased in the low-risk group (*P* < 0.001). Besides, expressions of HLA and checkpoint genes, such as CD274, CTLA4, LAG3 and TIGIT3, were upregulated in low-risk group. Additionally, highly expressed MGAT5 was validated in breast cancer tissues and cells. Downstream glycosylation products of MGAT5 were all increased in breast cancer.

**Conclusions:**

We identified a 19-glycogene signature for risk prediction of breast cancer patients. Patients in the low-risk group demonstrated a higher immune infiltration and better immunotherapy response. The validation of MGAT5 protein suggests a probable pathway and target for the development and treatment of breast cancer.

## Introduction

Breast cancer is one of the most common causes of morbidity and mortality in women worldwide (1). In addition to the traditional Tumor-Node-Metastasis (TNM) staging system, the treatment strategy of breast cancer is more dependent on its molecular typing characteristics, such as estrogen receptor (ER), progesterone receptor (PR), HER2, Ki67, and other risk factors like genomic markers (eg, BRCA1, BRCA2, and PIK3CA), and immunomarkers (eg, PD-L1) (2, 3). Based on these, we give patients chemotherapy, targeted therapy, endocrine therapy, radiotherapy and immunotherapy (4). In recent years, immunotherapy has also made great progress in the field of cancer therapy and has made some achievements in the treatment of breast cancer (5–7). Nevertheless, we still need to select appropriate patients for immunotherapy more accurately.

Breast cancer patients’ outcomes are erratic due to heterogeneity at the genetic, transcriptional, and posttranscriptional levels (8, 9). Even though the clinical events were identical, the underlying molecular and genetic processes were dissimilar; consequently, numerous studies exploited gene expression profiles to identify molecular characteristics that cross stage or grade categorization (10–13). This type of molecular categorization aids in the accurate prediction of breast cancer outcomes and treatment options.

As an important post-translational modification, glycosylation reflects a process in which monosaccharides or entire oligosaccharides (glycans) are enzymatically linked to specific amino acids of proteins (14–17). Glycosyltransferase, glycosidase, and sulfotransferase related genes are called “glycogenes” that participate in the regulation of glycosylation (18–20). Aberrant glycogenes expression or glycosylation actively contributes to tumor progression and is a key hallmark of cancer (14–17). Clinically, specific glycosylation of glycoprotein expression in blood are defined as tumor biomarkers (CA15-3, CA125 and CA19-9) (21–23). CA19-9 is a glycosylated sialic acid Lewis A, which is the most widely used serum tumor marker in pancreatic cancer and other gastroenterological cancers. In addition, other glycosylated structures and glycoproteins used in tumor diagnosis include prostate specific antigen (PSA), CEA, and CA72-4 (22, 24). Previous study reported that serum CEA, CA125 and CA15-3 levels was of great value in the management of breast cancer patients for they could be used as predictors and recurrence monitoring indicators (25). Furthermore, abnormal glycosylation affects the immune system’s perception of the tumor and can trigger immunosuppressive signals *via* glycan-binding receptors (26).

Using public transcriptome and survival data, we attempted to divide breast cancer patients into two risk groups using bioinformatics analysis. Following that, a 19-glycogene signature was discovered to successfully predict breast cancer patient outcomes. In addition, we looked at the immune microenvironment and checkpoint genes in different risk groups to identify potential immunotherapy patients. MGAT5, which synthesized the branching GlcNAc structures, was chosen for preliminary verification because it is an important glycosyltransferase that is overexpressed in breast cancer. Changing the content of glycosylation products is highly likely to regulate breast cancer’s biological activity.

## Methods

### Data from TCGA and GTEx databases

We recruited FPKM formatted sequencing data of 1104 female breast cancer samples and 113 normal mammary samples in TCGA database and another 80 female normal mammary tissues in the GTEx database from UCSC Xena website (https://xenabrowser.net/). FPKM refers to the fragments per kilobase of transcript per million fragments mapped, indicating that the calculation normalizes the transcript abundance by dividing it by the transcript length and total number of the aligned reads. The glycogene expression data was retrieved, and differentially expressed genes were examined using “Limma” R package. In addition, the follow-up time and survival status were documented. A total of 185 glycogenes were gathered from the glycogene database (GGDB, https://acgg.asia/ggdb2/) and prior publications (18).

### Establishment of the glycogene signature

First, we used univariate Cox regression analysis on differentially expressed genes (DEGs) to identify genes that were substantially associated to breast cancer overall survival (OS). To decrease the possibility of overfitting, we created a penalty function and employed the least absolute shrinkage and selection operator regression method to obtain a more accurate risk model. Finally, the glycogenes identified in the previous stage were subjected to the multivariate Cox regression analysis, and the resulting genes were utilized to create an independent gene prediction signature. The risk score of the gene signature is calculated using the following formula: Risk score = h (t, X) = h_0_(t) ×e∑ (coefi * Expri). Expri denotes gene expression, whereas h_0_(t) and coefi reflect the constant and coefficient determined from multivariate Cox regression analysis, respectively. The high- and low-risk groups were split by the median risk score and the Kaplan-Meier analysis and log-rank test were done to analyze their survival difference. ROC analysis was carried out to further assess the prognostic signature’s accuracy. In these investigations, the R packages “Survminer” and “survivalROC” were used.

### Immune infiltration profiles and immune checkpoint gene expression in different risk groups

By the unsupervised consensus cluster analysis, we calculated the tumor purity, immune scores, stromal scores, and estimate scores, and deduced the distribution of stromal and immune cells in tumor tissues (27). Our study adopted CIBERSORT algorithm to detect the relative percentage of 22 immune cells in each tumor tissue sample, using the LM22 signature matrix to run the algorithm under 1000 permutations (28). Then, we explored the relationships between risk scores and immune scores, immune infiltrating cells, and immune checkpoint genes’ abundance based on the single sample gene set enrichment analysis (29).

### GO and KEGG enrichment analysis

To further analyze the function of the glycogene signature, we performed Gene Ontology (GO) and Kyoto Encyclopedia of Genes and Genome (KEGG) pathway enrichment analysis (29). GO analysis included three categories, namely biological process (BP), cellular component (CC) and molecular function (MF). *P* < 0.05 was regarded as a meaningful threshold for functional pathway evaluation.

### Immunohistochemistry assay

Breast cancer samples were obtained immediately after surgery in the second affiliated hospital of Xi’an Jiaotong University (Xi’an, China). The formalin-fixed paraffin embedded tissues were prepared according to standard protocol. After antigen retrieval, sections were incubated with primary anti-MGAT5 antibody (1/50, Santa Cruz Biotechnology, USA) or anti-PHA-L (phaseolus vulgavis leucoagglutinin) lectin (1/100, Vector Laboratories, USA) overnight at 4°C, followed by the incubation with the horseradish peroxidase-conjugated antibody. PHA-L recognizes branching GlcNAc structures. The slides were further proceeded by DAB visualization kit (DAB-0031; Maixin_Bio, China), and counterstained with hematoxylin. Finally, images were captured by a slide scanner (Science; WinMedic, China). This study was approved by the institutional review board of the second affiliated hospital of Xi’an Jiaotong University, with the written informed consent from the corresponding patients.

### Cell culture

Immortalized human mammary epithelial cell line MCF10A and human BC cell lines MDA‐MB‐231, MCF7, MDA‐MB‐468, and MDA‐MB‐453 were purchased from the American Type Culture Collection (30). MCF10A cells were cultured in Dulbecco’s modified Eagle’s medium (DMEM)/F12 complete medium (Gibco; Thermo Fisher Scientific) supplemented with insulin (10 μg/mL), cholera toxin (100 ng/mL; Sigma‐Aldrich), hydrocortisone (0.5 mg/mL), epidermal growth factor (EGF; 20 ng/mL). Human breast cancer cell lines were cultured in DMEM (HyClone, GE Healthcare). All the medium were supplemented with 10% fetal bovine serum (Biological Industries), penicillin (100 units/mL), and streptomycin (100 μg/mL). All cell lines were grown at 37°C in 5% CO_2_.

### Western blotting analysis

We prepared the whole cell lysates using RIPA lysis buffer with protease inhibitor cocktail. Protein concentrations of lysates were measured before being boiled in SDS loading buffer. Equal amounts of protein samples were loaded onto 10% SDS-PAGE, and transferred to nitrocellulose membranes. The rest steps of western blotting were then performed following standard protocol. The primary antibodies against MGAT5 (1/1000, Santa Cruz Biotechnology, USA), GAPDH (1/1,000, Santa Cruz Biotechnology, USA) and HRP-conjugated anti-rabbit or anti-mouse antibodies (1/5,000) were used. The visualization was done with the Luminescent Imaging System (Tanon, China).

### Statistical analysis

Unpaired Student t-test was performed for two independent variables. Kaplan-Meier analysis with log-rank test was used for survival probability comparison. In addition, univariate and multivariate Cox regression were done to determine the independent prognostic factors. Statistical analysis was performed by Bioconductor/R. For all analysis, *P*< 0.05 is considered statistically significant.

## Results

### Identification of differentially expressed glycogenes between breast cancer and normal tissues

**Figure 1:**
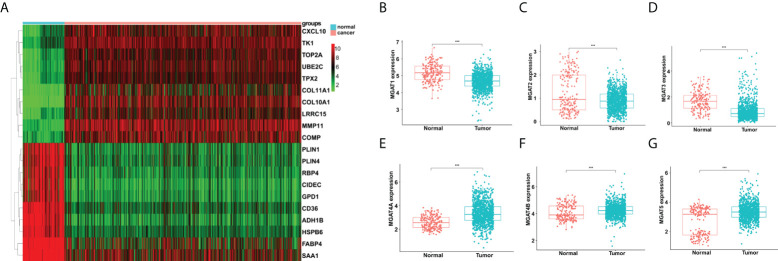
Analyses of gene expression differences in breast cancer and normal mammary tissues from the TCGA and GTEx databases. **(A)** Heatmap of top 20 DEGs. Comparisons of MGAT1 **(B)**, MGAT2 **(C)**, MGAT3 **(D)**, MGAT4A **(E)**, MGAT4B **(F)** and MGAT5 **(G)** expressions between breast cancer and normal tissues. *** means p<0.001.

We collected expression profiles from the GTEx and TCGA databases for 192 female normal mammary tissue and 1092 female breast cancer samples. The heatmap displays the top 20 differentially expressed genes (DEGs) in breast cancer *vs*. normal tissues (**Figure 1A**). While, the top 100 DEGs are also listed in **Table 1**. Next, we performed Go analysis to explore the enrichment of the DEGs in cellular component (CC), molecular function (MF) and biological process (BP). DEGs were enriched in terms of ncRNA processing, rRNA processing, DNA replication, cell-substrate junction, cell-substrate adherens junction, protein ligase binding, cadherin binding, and GTPase binding (**Supplementary Figure 1A**). DEGs were enriched pathways like Cell cycle, Malignant tumors, Rap1, MAPK, p53 signaling pathways and N-glycan biosynthesis, according to KEGG analysis (**Supplementary Figure 1B**).

**Table 1:** Identification of top 100 DEGs between breast cancer and normal tissues.

	Up-regulated	Down-regulated
**DEGs**	COL10A1, MMP11, UBE2C, COMP, COL11A1, TOP2A, TPX2, LRRC15, CXCL10, TK1, MYBL2, RPL41P1, GJB2, UBE2T, S100P, CKS2, S100A14, TFF1, CDC20, MMP9, IGHG4, NUSAP1, IFI6, COL1A1, CEACAM6, BIRC5, NEK2, LINC01614, MISP, RRM2, ASF1B, EEF1A2, CST1, CXCL9, CTXN1, AGR2, BGN, INHBA, ISG15, ZWINT, SDC1, PYCR1, NKAIN1, CRABP2, CCNB1, CENPF, MMP13, PAFAH1B3, HIST1H4H, PITX1	FABP4, ADH1B, CIDEC, PLIN1, CD36, SAA1, HSPB6, GPD1, RBP4, PLIN4, GPX3, ADIPOQ, FHL1, BTNL9, CRYAB, LIPE, LEP, CD300LG, SAA2, CCL14, HBB, CHRDL1, TNXB, CFD, LPL, AQP7, PDK4, HBA2, TRARG1, LYVE1, SCARA5, MYH11, TIMP4, SFRP1, CIDEA, KRT14, CLDN5, CAVIN2, FMO2, EEF1G, CLEC3B, C2orf40, G0S2, ITGA7, AKR1C2, KRT5, APOD, CA4, IGFBP6, PPP1R1A

DEGs, differentially expressed genes.

Then we obtained 185 glycogenes from the glycogene database (GGDB, https://acgg.asia/ggdb2/) (**Supplementary Table 1**). A total of 131 glycogenes were discovered to be expressed differentially in breast cancer and normal tissues (**Supplementary Table 2**). The comparison of MGAT family protein expression is shown in **Figure 1**. In breast cancer, MGAT1, MGAT2 and MGAT3 were downregulated, but MGAT4A, MGAT4B, and MGAT5 were increased (**Figures 1B–G**).

### Identification of glycogenes correlated to prognosis of breast cancer patients

**Figure 2:**
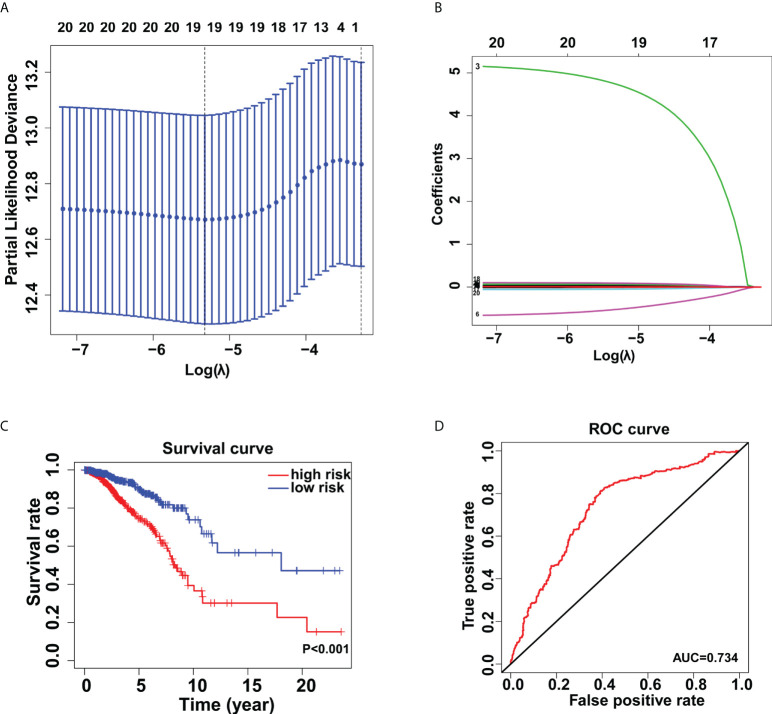
Establishment of the glycogene signature in breast cancer. Lasso regression analysis **(A, B)** for identification of the glycogene signature. **(C)** Kaplan-Meier analysis of overall survival rates in low- and high-risk groups. **(D)** Validation of the glycogene signature’s predictive performance by the receiver operating characteristic (ROC) curve.

Through univariate Cox regression analysis, twenty glycogenes, ALG2, ALG3, B3GNT3, B4GALNT2, C1GALT1C1, CHST10, DPAGT1, FUT11, FUT3, FUT7, GALNT1, GALNT9, HS3ST5, HS6ST2, NDST4, RFNG, SLC35A2, SLC35A3, ST3GAL1, and ST6GALNAC4, were found to be correlated with the OS of breast cancer patients. Then, the LASSO regression analysis was applied to identify relevant genes with a minimum criterion optimal λ value (**Figures 2A, B**). Lastly, multivariate Cox regression analysis was performed and 19 independent prognostic genes were obtained to construct the model. The formula used for risk score computation was as follows: 0.011×ALG2 expression + 0.015×ALG3 expression + 0.010×B3GNT3 expression + 0.014×C1GALT1C1 expression + (-0.049)×CHST10 expression + 0.009×DPAGT1 expression + 0.091×FUT11 expression + 0.018×FUT3 expression + (-0.524)×FUT7 expression + 0.007×GALNT1 expression + 4.741×GALNT9 expression + 0.063×HS3ST5 expression + 0.016×HS6ST2 expression + 0.041×NDST4 expression+(-0.005) × RFNG expression + 0.009×SLC35A2 expression + 0.013×SLC35A3 expression + 0.011×ST3GAL1 expression + 0.026×ST6GALNAC4 expression. Based on the medium risk score, patients were separated into high- and low- risk groups, which showed distinct overall survival (*P*< 0.001) (**Figure 2C**). This glycogene signature was further verified by receiver operating characteristic curve (ROC) (AUC=0.734) (**Figure 2D**).

### Immune cell infiltration of two risk groups in breast cancer

**Figure 3:**
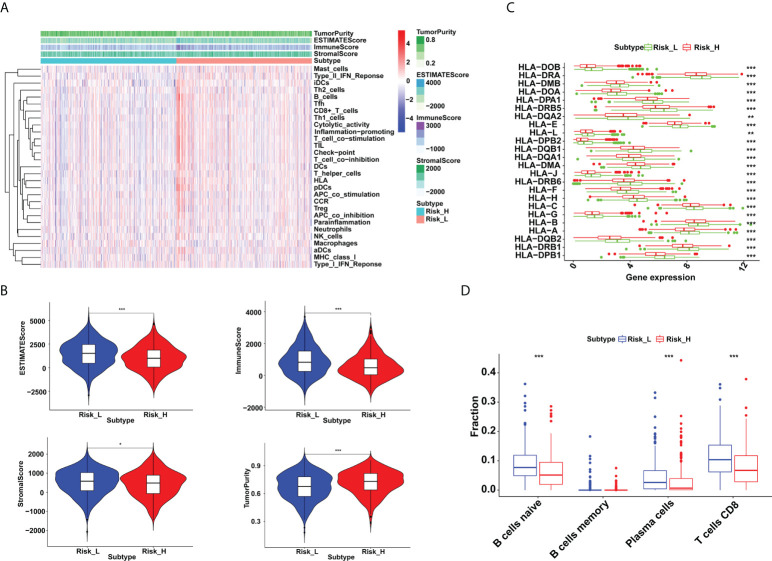
Immune microenvironment differences between low- and high-risk groups in breast cancer. **(A)** The distribution of immunocytes in the high- and low-risk groups. **(B)** Higher immune, stromal, and ESTIMATE scores, and lower tumor purity were exhibited in the low-risk group (*P < 0.05 and ***P < 0.001). **(C)** The HLA-related genes’ expressions were upregulated in the low-risk group (**P < 0.01 and ***P < 0.001). **(D)** Proportion of naive B cells, plasma cells and CD8^+^ T cells were increased in the low-risk group (***P < 0.001).

The tumor microenvironment estimations were made up of stromal, immune, estimate, and tumor purity scores (**Figure 3A**). Our findings revealed that patients in the low-risk group had higher stromal, immunological, and estimation scores, and reduced tumor purity (**Figure 3B**). We also demonstrated a statistically significant relationship between risk score and HLA-related gene expression. As demonstrated in **Figure 3C**, higher HLA-related genes’ abundance was observed in the low-risk group than the high-risk group. Furthermore, we found that the proportion of naive B cells, plasma cells, and CD8^+^ T cells increased in the low-risk group (*P* < 0.001) (**Figure 3D**).

### Prediction of immunotherapy response based on the glycogene signature in breast cancer

**Figure 4:**
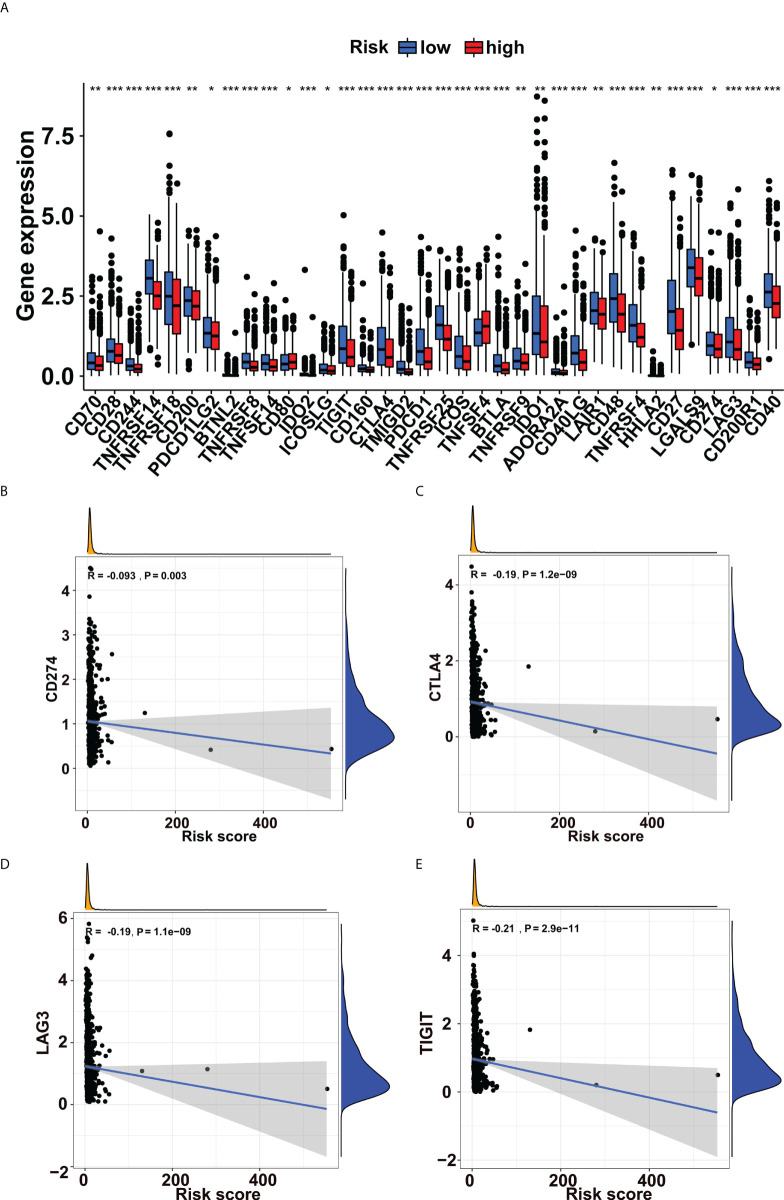
Immune checkpoint genes’ expressions increased in the low-risk group. **(A)** Expression levels of immune checkpoint genes in the low- and high-risk groups. The expressions of **(B)** CD274, **(C)** CTLA4, **(D)** LAG3, and **(E)** TIGIT were negatively correlated with risk scores in breast cancer. *p<0.05, **p<0.01, ***p<0.001.

Immune checkpoint blockade (ICB) is a novel therapeutic technique that offers fresh hope for cancer treatment. More and more checkpoint genes are being identified as possible therapeutic targets. In this study, we tried to use the glycogene signature to evaluate the ICB response. We revealed that immune checkpoint genes such as CD274, CTLA4, LAG3 and TIGIT were more expressed in low-risk groups (**Figure 4A**). Besides, we explored the correlations between immune checkpoint gene expression and risk scores in breast cancer. We found that they were all adversely correlated (**Figures 4B–E**, **Supplementary Figure 2**).

### Validation of a glycosyltransferase, MGAT5 protein in breast cancer

**Figure 5:**
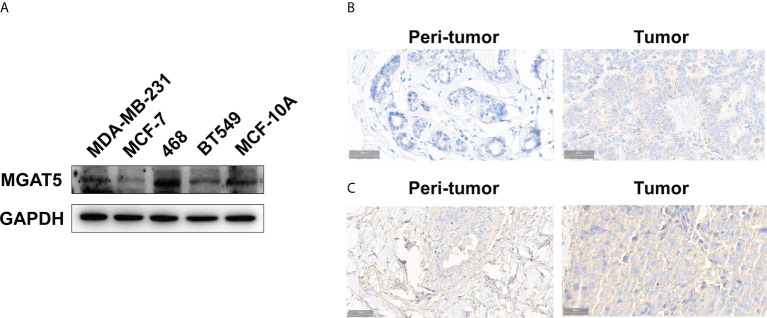
The expression of MGAT5 and branching GlcNAc structures in breast cancer. **(A)** The protein levels of MGAT5 in breast cancer cell lines (western blotting). **(B)** The MGAT5 level was upregulated in breast cancer (immunohistochemistry). **(C)** The expression of branching GlcNAc structures was increased in breast cancer samples (immunohistochemistry).

Furthermore, we gathered breast cancer tissue samples to validate the results generated from the public databases. The findings of the IHC staining revealed that the MGAT5 protein was high expressed in breast cancer cells (**Figure 5A**). Meanwhile, we examined its expression in breast cancer tissues. As shown in **Figure 5B**, MGAT5 expression was also increased in breast cancer tissues.

Besides, we tested the levels of MGAT5 glycan products in breast cancer tissues. Our results revealed that PHA-L labeled signal was higher in breast cancer tissues, which indicated increased glycan products modified by MGAT5 (**Figure 5C**).

## Discussion

Based on the role of glycosylation in tumor transformation and the emergence of new typing methods in cancer staging, we identified a novel 19-glycogene signature for clinical outcomes, immune infiltration, and immunotherapy response prediction in breast cancer patients in this study. The novel finding is that patients in the low-risk group may have better immunotherapy response because of higher immune infiltration and checkpoint gene abundance. This provides a novel tool for the selection of potential patients for immunotherapy.

Abnormal glycosylation modification is closely related to tumor occurrence, proliferation, invasion, metastasis, and immune escape (31–34). Protein glycosylation modification can help immune cells locate and migrate correctly. In the present study, we did not explore the role of certain glycogene in cancer immune microenvironment or cancer development. But, according to the relationship between these genes’ expression and survival probabilities, we separated breast cancer patients into two groups with distinct immune infiltration and checkpoints gene expressions. The 19-glycogene signature demonstrate that proportion of naive B cells, plasma cells and CD8^+^ T cells increased in the low-risk group, which indicated these patients may have better immunotherapy response. Previous studies had similar results that showed higher immune infiltration in the low-risk group (35). The high density of cytotoxic T lymphocytes, natural killer cells, B lymphocytes and dendritic cells indicate a tumor microenvironment with an active immune reaction and favorable prognoses in cancer patients (36, 37). CD8^+^ T cells are important immune cells which recognize and clear tumor cells and thereby associated with improved survival in cancer patients (38). The relationships between these immune cells and glycosylation in breast cancers is worthy of further research. It is worth noting that HLA related genes’ expressions were all upregulated in low-risk group in this study. Histocompatibility complex participates in the recognition and presentation of tumor-associated antigens through T lymphocytes and dendritic cells, thus producing adaptive anti-cancer immune responses (39, 40). MHC downregulation is observed in cancers and promotes tumor cell immune escape, which results in immunotherapy resistance and cancer progression (41). Thus, a novel tool to predict the patients with high MHC abundance is important.

In the past decades, great advances have been made in the targeted therapy, especially in breast cancer and lung cancer. Trastuzumab, pertuzumab, small molecular tyrosine kinase inhibitor (TKI) and antibody-drug conjugate (ADC) have significantly improved the survival of HER-positive breast cancer patients (42, 43). Also, cancer immunotherapy has made rapid development. Immune checkpoint blockade (ICB) therapy has obvious effects on many cancers. In this study, the glycogene signature helped to identify potential patients for immunotherapy according to the checkpoint gene expressions. In our study, the famous PD-L1(CD274) was high expressed in the low-risk groups. PD-L1 inhibitor, atezolizumab, is recommended for the treatment of advanced triple negative breast cancer (44, 45). These evidences indicate that breast cancer patients at low risk may have good response to PD-L1 inhibitor. However, these results need more validation in clinical practice or greater cohort. Besides PD-L1, CTLA4, LAG3, TIGIT and other checkpoint genes, were all showing high expression in the low-risk group. These checkpoint genes were well explored and were potential targets in the ICB therapy of several solid tumors (46). Low risk based on this glycogene signature may help guide clinical practice. Although many new signatures have been used to classify breast cancer and help to judge the effect of immunotherapy, we first used glycogenes to construct a risk model, which partly verified the relationship between glycosylation and tumor immunity, and provided evidence for our later verification.

MGAT5 is one member of the glycosyltransferase family that adds the beta-1,6-N-acetylglucosamine to the alpha-linked mannose of biantennary N-linked oligosaccharides. In addition to its glycosyltransferase function, MGAT5 is involved in tumor transformation. MGAT5 regulates mesenchymal markers, growth factor receptors, and even immune cell infiltration to influence the malignant transformation and tumor metastasis of cells (47–50). In present study, we validated the increased MGAT5 protein and its glycosylation products in breast cancer. This finding, while preliminary, suggests that MGAT5 may participate in the development of breast cancer through itself or its modified products. However, further research should be undertaken to investigate the specific mechanisms.

Because this is a bioinformatics analysis with preliminary validation in breast cancer, additional validation is required. The main limitation of this study is that it is the data mining of glycogenes gene expression to construct signature. It is partly dependent on the original data’ authenticity and accuracy. Whether the results are robust still needs to be verified by more samples. In addition, this classification lacks a specific risk boundary value to determine whether patients are high- or low-risk group. We do not know whether they are candidates for immunotherapy. Therefore, similar studies and clinical validation in multiple centers are required to establish one or more cut-off values to guide clinical practice.

## Conclusions

In this study, we successfully identified a novel 19-glycogene signature, based on which breast cancer patients were separated into low- and high-risk groups. Patients at low risk have better prognosis and may have good response of immunotherapy. Abnormal glycosylation of key protein by MGAT5 may explain the mechanism of breast cancer development.

## Data availability statement

The datasets presented in this study can be found in online repositories. The names of the repository/repositories and accession number(s) can be found in the article/**Supplementary Material**.

## Author contributions

Conception and design: HK, FG, and ZD. Collection and assembly of data: SL, ZT, HC, QM, and XZ. Data analysis and interpretation: SL, ZT, JW, HC, and LD. Manuscript writing: SL, QM, and XZ. Manuscript revision: HC and SL. All authors contributed to the article and approved the submitted version.

## Funding

This study was supported by the International Science and Technology Cooperation Program Project of Shaanxi Province, China (2018KW-058), and the Fundamental Research Funds for the Central Universities of China (xzy012021062).

## Acknowledgments

We acknowledge the data support of the TCGA and GTEx databases, as well as R packages’ developers and providers.

## Conflict of interest

The authors declare that the research was conducted in the absence of any commercial or financial relationships that could be construed as a potential conflict of interest.

## Publisher’s note

All claims expressed in this article are solely those of the authors and do not necessarily represent those of their affiliated organizations, or those of the publisher, the editors and the reviewers. Any product that may be evaluated in this article, or claim that may be made by its manufacturer, is not guaranteed or endorsed by the publisher.

## Supplementary material

Supplementary Figure 1 Functional enrichment analysis of differentially expressed genes between breast cancer and normal tissues. **(A)** GO analysis for the DEGs. **(B)** KEGG pathways enriched for the DEGs. DEGs, differentially expressed genes.

Supplementary Figure 2 Correlations between immune checkpoint genes’ expressions and risk scores in breast cancer.
