# Supplementary figures and images for: Identification of glycogene signature as a tool to predict the clinical outcome and immunotherapy response in breast cancer
Source: Front Oncol. 2022 Sep 14;12:854284. doi: 10.3389/fonc.2022.854284 (PMC9515430; doi:10.3389/fonc.2022.854284)

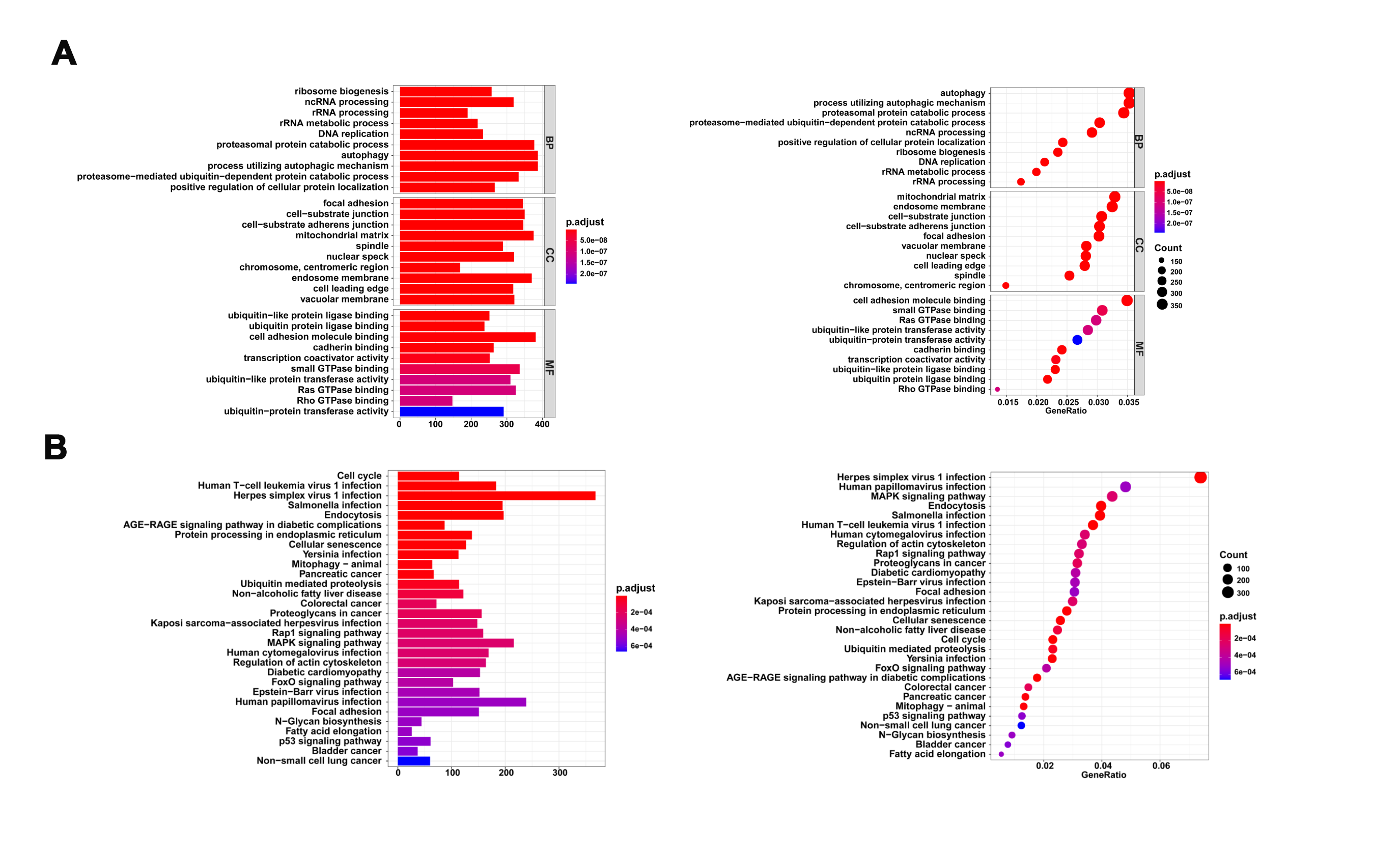

Supplement: Supplementary Figure 1 — Functional enrichment analysis of differentially expressed genes between breast cancer and normal tissues. (A) GO analysis for the DEGs. (B) KEGG pathways enriched for the DEGs. DEGs, differentially expressed genes. [file Image_1.tif]

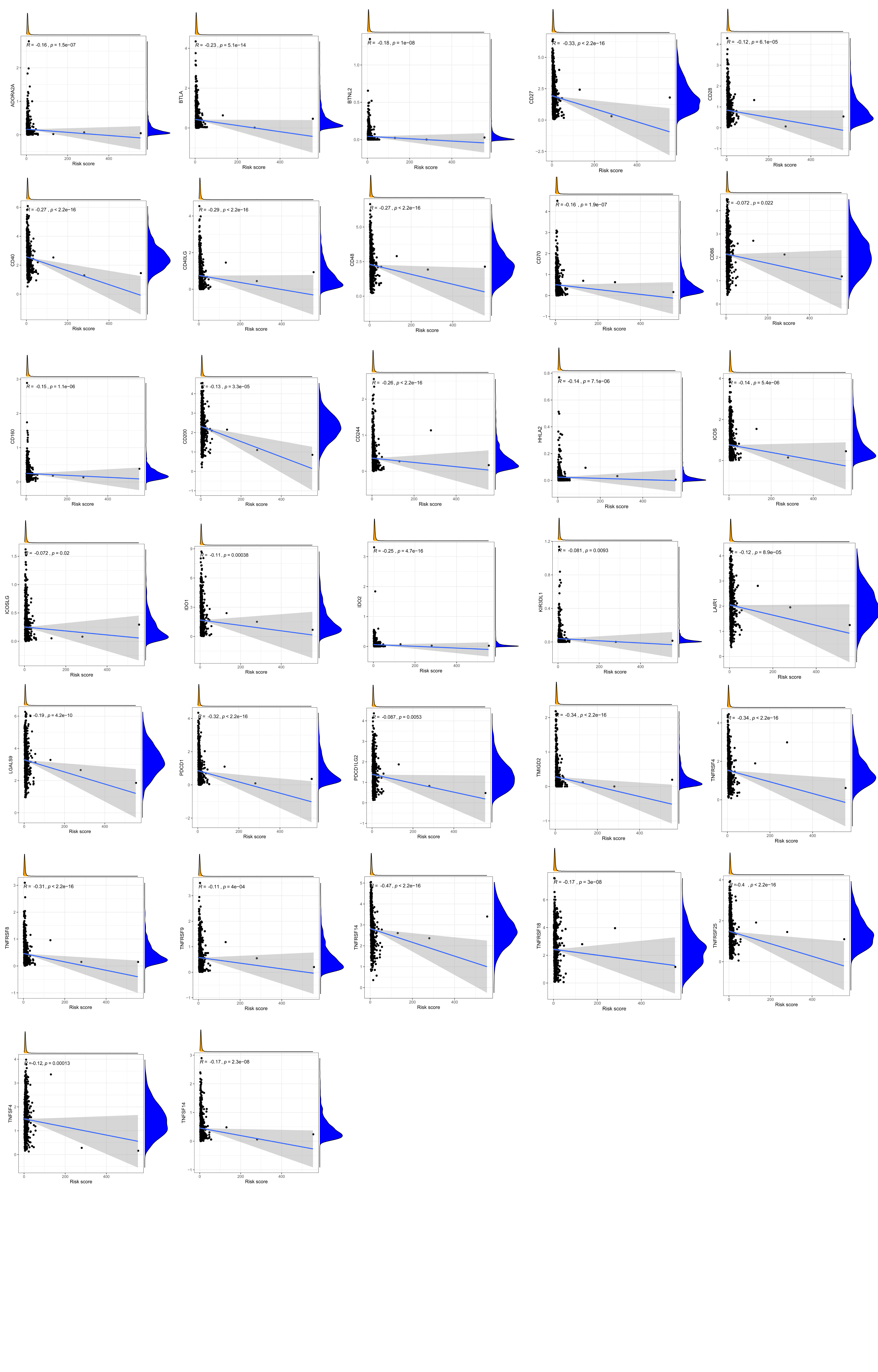

Supplement: Supplementary Figure 2 — Correlations between immune checkpoint genes’ expressions and risk scores in breast cancer. [file Image_2.tif]
