# Supplementary material for: Identification of glycogene signature as a tool to predict the clinical outcome and immunotherapy response in breast cancer
Source: Front Oncol. 2022 Sep 14;12:854284. doi: 10.3389/fonc.2022.854284 (PMC9515430; doi:10.3389/fonc.2022.854284)
Supplement: Supplementary file 3 [file Table_1.docx]

**Supplementary Table 1 Details of 185 glycogenes obtained from the glycogene database**

| **Gene symbols** | **Full names** |
| --- | --- |
| A4GALT | alpha 1,4-galactosyltransferase |
| A4GNT | alpha-1,4-N-acetylglucosaminyltransferase |
| ABO | ABO blood group (transferase A, alpha 1-3-N-acetylgalactosaminyltransferase; transferase B, alpha 1-3-galactosyltransferase) |
| ALG1 | asparagine-linked glycosylation 1, beta-1,4-mannosyltransferase homolog (S. cerevisiae) |
| ALG10 | asparagine-linked glycosylation 10, alpha-1,2-glucosyltransferase homolog (S. pombe) |
| ALG11 | UTP14, U3 small nucleolar ribonucleoprotein, homolog C (yeast); asparagine-linked glycosylation 11, alpha-1,2-mannosyltransferase homolog (yeast) |
| ALG12 | asparagine-linked glycosylation 12, alpha-1,6-mannosyltransferase homolog (S. cerevisiae) |
| ALG13 | asparagine-linked glycosylation 13 homolog (S. cerevisiae) |
| ALG14 | asparagine-linked glycosylation 14 homolog (S. cerevisiae) |
| ALG2 | asparagine-linked glycosylation 2, alpha-1,3-mannosyltransferase homolog (S. cerevisiae) |
| ALG3 | asparagine-linked glycosylation 3, alpha-1,3- mannosyltransferase homolog (S. cerevisiae) |
| ALG5 | asparagine-linked glycosylation 5, dolichyl-phosphate beta-glucosyltransferase homolog (S. cerevisiae) |
| ALG6 | asparagine-linked glycosylation 6, alpha-1,3-glucosyltransferase homolog (S. cerevisiae) |
| ALG8 | asparagine-linked glycosylation 8, alpha-1,3-glucosyltransferase homolog (S. cerevisiae) |
| ALG9 | asparagine-linked glycosylation 9, alpha-1,2-mannosyltransferase homolog (S. cerevisiae) |
| B3GALNT1 | beta-1,3-N-acetylgalactosaminyltransferase 1 (globoside blood group) |
| B3GALNT2 | beta-1,3-N-acetylgalactosaminyltransferase 2 |
| B3GALT1 | UDP-Gal:betaGlcNAc beta 1,3-galactosyltransferase, polypeptide 1 |
| B3GALT2 | UDP-Gal:betaGlcNAc beta 1,3-galactosyltransferase, polypeptide 2 |
| B3GALT4 | UDP-Gal:betaGlcNAc beta 1,3-galactosyltransferase, polypeptide 4 |
| B3GALT5 | UDP-Gal:betaGlcNAc beta 1,3-galactosyltransferase, polypeptide 5 |
| B3GALT6 | UDP-Gal:betaGal beta 1,3-galactosyltransferase polypeptide 6 |
| B3GALTL | beta 1,3-galactosyltransferase-like |
| B3GAT1 | beta-1,3-glucuronyltransferase 1 (glucuronosyltransferase P) |
| B3GAT2 | beta-1,3-glucuronyltransferase 2 (glucuronosyltransferase S) |
| B3GAT3 | beta-1,3-glucuronyltransferase 3 (glucuronosyltransferase I) |
| B3GNT1 | UDP-GlcNAc:betaGal beta-1,3-N-acetylglucosaminyltransferase 1; UDP-GlcNAc:betaGal beta-1,3-N-acetylglucosaminyltransferase 2 |
| B3GNT3 | UDP-GlcNAc:betaGal beta-1,3-N-acetylglucosaminyltransferase 3 |
| B3GNT4 | UDP-GlcNAc:betaGal beta-1,3-N-acetylglucosaminyltransferase 4 |
| B3GNT5 | UDP-GlcNAc:betaGal beta-1,3-N-acetylglucosaminyltransferase 5 |
| B3GNT6 | UDP-GlcNAc:betaGal beta-1,3-N-acetylglucosaminyltransferase 6 (core 3 synthase) |
| B3GNT7 | UDP-GlcNAc:betaGal beta-1,3-N-acetylglucosaminyltransferase 7 |
| B3GNT8 | UDP-GlcNAc:betaGal beta-1,3-N-acetylglucosaminyltransferase 8 |
| B4GALNT1 | beta-1,4-N-acetyl-galactosaminyl transferase 1 |
| B4GALNT2 | beta-1,4-N-acetyl-galactosaminyl transferase 2 |
| B4GALNT3 | beta-1,4-N-acetyl-galactosaminyl transferase 3 |
| B4GALNT4 | beta-1,4-N-acetyl-galactosaminyl transferase 4 |
| B4GALT1 | UDP-Gal:betaGlcNAc beta 1,4- galactosyltransferase, polypeptide 1 |
| B4GALT2 | UDP-Gal:betaGlcNAc beta 1,4- galactosyltransferase, polypeptide 2 |
| B4GALT3 | UDP-Gal:betaGlcNAc beta 1,4- galactosyltransferase, polypeptide 3 |
| B4GALT4 | UDP-Gal:betaGlcNAc beta 1,4- galactosyltransferase, polypeptide 4 |
| B4GALT5 | UDP-Gal:betaGlcNAc beta 1,4- galactosyltransferase, polypeptide 5 |
| B4GALT6 | UDP-Gal:betaGlcNAc beta 1,4- galactosyltransferase, polypeptide 6 |
| B4GALT7 | xylosylprotein beta 1,4-galactosyltransferase, polypeptide 7 (galactosyltransferase I) |
| C1GALT1 | core 1 synthase, glycoprotein-N-acetylgalactosamine 3-beta-galactosyltransferase, 1 |
| C1GALT1C1 | C1GALT1-specific chaperone 1 |
| CHPF | chondroitin polymerizing factor |
| CHPF2 | chondroitin sulfate glucuronyltransferase |
| CHST1 | carbohydrate (keratan sulfate Gal-6) sulfotransferase 1 |
| CHST10 | carbohydrate sulfotransferase 10 |
| CHST11 | carbohydrate (chondroitin 4) sulfotransferase 11 |
| CHST12 | carbohydrate (chondroitin 4) sulfotransferase 12 |
| CHST13 | carbohydrate (chondroitin 4) sulfotransferase 13 |
| CHST14 | carbohydrate (N-acetylgalactosamine 4-0) sulfotransferase 14 |
| CHST15 | carbohydrate (N-acetylgalactosamine 4-sulfate 6-O) sulfotransferase 15 |
| CHST2 | carbohydrate (N-acetylglucosamine-6-O) sulfotransferase 2 |
| CHST3 | carbohydrate (chondroitin 6) sulfotransferase 3 |
| CHST4 | carbohydrate (N-acetylglucosamine 6-O) sulfotransferase 4 |
| CHST5 | carbohydrate (N-acetylglucosamine 6-O) sulfotransferase 5 |
| CHST6 | carbohydrate (N-acetylglucosamine 6-O) sulfotransferase 6 |
| CHST7 | carbohydrate (N-acetylglucosamine 6-O) sulfotransferase 7 |
| CHST8 | carbohydrate (N-acetylgalactosamine 4-0) sulfotransferase 8 |
| CHST9 | carbohydrate (N-acetylgalactosamine 4-0) sulfotransferase 9 |
| CHSY1 | chondroitin sulfate synthase 1 |
| CHSY3 | chondroitin sulfate synthase 3 |
| CSGALNACT1 | chondroitin sulfate N-acetylgalactosaminyltransferase 1 |
| CSGALNACT2 | chondroitin sulfate N-acetylgalactosaminyltransferase 2; novel protein similar to chondroitin sulfate GalNAcT-2 (GALNACT-2) |
| DPAGT1 | dolichyl-phosphate (UDP-N-acetylglucosamine) N-acetylglucosaminephosphotransferase 1 (GlcNAc-1-P transferase) |
| DPM1 | dolichyl-phosphate mannosyltransferase polypeptide 1, catalytic subunit |
| DPM2 | dolichyl-phosphate mannosyltransferase polypeptide 2, regulatory subunit |
| DPM3 | dolichyl-phosphate mannosyltransferase polypeptide 3 |
| EXT1 | exostoses (multiple) 1 |
| EXT2 | exostoses (multiple) 2 |
| EXTL1 | exostoses (multiple)-like 1 |
| EXTL2 | exostoses (multiple)-like 2 |
| EXTL3 | exostoses (multiple)-like 3 |
| FKRP | fukutin related protein |
| FKTN | fukutin |
| FUT1 | fucosyltransferase 1 (galactoside 2-alpha-L-fucosyltransferase, H blood group) |
| FUT10 | fucosyltransferase 10 (alpha (1,3) fucosyltransferase) |
| FUT11 | fucosyltransferase 11 (alpha (1,3) fucosyltransferase) |
| FUT2 | fucosyltransferase 2 (secretor status included) |
| FUT3 | fucosyltransferase 3 (galactoside 3(4)-L-fucosyltransferase, Lewis blood group) |
| FUT4 | fucosyltransferase 4 (alpha (1,3) fucosyltransferase, myeloid-specific) |
| FUT5 | fucosyltransferase 5 (alpha (1,3) fucosyltransferase) |
| FUT6 | fucosyltransferase 6 (alpha (1,3) fucosyltransferase) |
| FUT7 | fucosyltransferase 7 (alpha (1,3) fucosyltransferase) |
| FUT8 | fucosyltransferase 8 (alpha (1,6) fucosyltransferase) |
| FUT9 | fucosyltransferase 9 (alpha (1,3) fucosyltransferase) |
| GAL3ST1 | galactose-3-O-sulfotransferase 1 |
| GAL3ST2 | galactose-3-O-sulfotransferase 2 |
| GAL3ST3 | galactose-3-O-sulfotransferase 3 |
| GAL3ST4 | galactose-3-O-sulfotransferase 4 |
| GALNT1 | UDP-N-acetyl-alpha-D-galactosamine:polypeptide N-acetylgalactosaminyltransferase 13 (GalNAc-T13); UDP-N-acetyl-alpha-D-galactosamine:polypeptide N-acetylgalactosaminyltransferase 1 (GalNAc-T1) |
| GALNT10 | UDP-N-acetyl-alpha-D-galactosamine:polypeptide N-acetylgalactosaminyltransferase 10 (GalNAc-T10) |
| GALNT11 | UDP-N-acetyl-alpha-D-galactosamine:polypeptide N-acetylgalactosaminyltransferase 11 (GalNAc-T11) |
| GALNT12 | UDP-N-acetyl-alpha-D-galactosamine:polypeptide N-acetylgalactosaminyltransferase 12 (GalNAc-T12) |
| GALNT14 | UDP-N-acetyl-alpha-D-galactosamine:polypeptide N-acetylgalactosaminyltransferase 14 (GalNAc-T14) |
| GALNT15 | UDP-N-acetyl-alpha-D-galactosamine:polypeptide N-acetylgalactosaminyltransferase-like 2 |
| GALNT2 | UDP-N-acetyl-alpha-D-galactosamine:polypeptide N-acetylgalactosaminyltransferase 2 (GalNAc-T2) |
| GALNT3 | UDP-N-acetyl-alpha-D-galactosamine:polypeptide N-acetylgalactosaminyltransferase 3 (GalNAc-T3) |
| GALNT4 | UDP-N-acetyl-alpha-D-galactosamine:polypeptide N-acetylgalactosaminyltransferase 4 (GalNAc-T4) |
| GALNT5 | UDP-N-acetyl-alpha-D-galactosamine:polypeptide N-acetylgalactosaminyltransferase 5 (GalNAc-T5) |
| GALNT6 | UDP-N-acetyl-alpha-D-galactosamine:polypeptide N-acetylgalactosaminyltransferase 6 (GalNAc-T6) |
| GALNT7 | UDP-N-acetyl-alpha-D-galactosamine:polypeptide N-acetylgalactosaminyltransferase 7 (GalNAc-T7) |
| GALNT8 | UDP-N-acetyl-alpha-D-galactosamine:polypeptide N-acetylgalactosaminyltransferase 8 (GalNAc-T8) |
| GALNT9 | UDP-N-acetyl-alpha-D-galactosamine:polypeptide N-acetylgalactosaminyltransferase 9 (GalNAc-T9) |
| GBGT1 | globoside alpha-1,3-N-acetylgalactosaminyltransferase 1 |
| GCNT1 | glucosaminyl (N-acetyl) transferase 1, core 2 (beta-1,6-N-acetylglucosaminyltransferase) |
| GCNT2 | glucosaminyl (N-acetyl) transferase 2, I-branching enzyme (I blood group) |
| GCNT3 | glucosaminyl (N-acetyl) transferase 3, mucin type |
| GCNT4 | glucosaminyl (N-acetyl) transferase 4, core 2 (beta-1,6-N-acetylglucosaminyltransferase) |
| GYLTL1B | glycosyltransferase-like 1B |
| HAS1 | hyaluronan synthase 1 |
| HAS2 | hyaluronan synthase 2 |
| HAS3 | hyaluronan synthase 3 |
| HS2ST1 | heparan sulfate 2-O-sulfotransferase 1 |
| HS3ST1 | heparan sulfate (glucosamine) 3-O-sulfotransferase 1 |
| HS3ST2 | heparan sulfate (glucosamine) 3-O-sulfotransferase 2 |
| HS3ST3A1 | heparan sulfate (glucosamine) 3-O-sulfotransferase 3A1 |
| HS3ST3B1 | heparan sulfate (glucosamine) 3-O-sulfotransferase 3B1 |
| HS3ST4 | heparan sulfate (glucosamine) 3-O-sulfotransferase 4 |
| HS3ST5 | heparan sulfate (glucosamine) 3-O-sulfotransferase 5 |
| HS6ST1 | heparan sulfate 6-O-sulfotransferase 1 |
| HS6ST2 | heparan sulfate 6-O-sulfotransferase 2 |
| HS6ST3 | heparan sulfate 6-O-sulfotransferase 3 |
| LARGE | like-glycosyltransferase |
| LFNG | LFNG O-fucosylpeptide 3-beta-N-acetylglucosaminyltransferase |
| MFNG | MFNG O-fucosylpeptide 3-beta-N-acetylglucosaminyltransferase |
| MGAT1 | mannosyl (alpha-1,3-)-glycoprotein beta-1,2-N-acetylglucosaminyltransferase |
| MGAT2 | mannosyl (alpha-1,6-)-glycoprotein beta-1,2-N-acetylglucosaminyltransferase |
| MGAT3 | mannosyl (beta-1,4-)-glycoprotein beta-1,4-N-acetylglucosaminyltransferase |
| MGAT4A | mannosyl (alpha-1,3-)-glycoprotein beta-1,4-N-acetylglucosaminyltransferase, isozyme A |
| MGAT4B | mannosyl (alpha-1,3-)-glycoprotein beta-1,4-N-acetylglucosaminyltransferase, isozyme B |
| MGAT5 | mannosyl (alpha-1,6-)-glycoprotein beta-1,6-N-acetyl-glucosaminyltransferase; hypothetical LOC151162 |
| MGAT5B | mannosyl (alpha-1,6-)-glycoprotein beta-1,6-N-acetyl-glucosaminyltransferase, isozyme B |
| NDST1 | N-deacetylase/N-sulfotransferase (heparan glucosaminyl) 1 |
| NDST2 | N-deacetylase/N-sulfotransferase (heparan glucosaminyl) 2 |
| NDST3 | N-deacetylase/N-sulfotransferase (heparan glucosaminyl) 3 |
| NDST4 | N-deacetylase/N-sulfotransferase (heparan glucosaminyl) 4 |
| OGT | O-linked N-acetylglucosamine (GlcNAc) transferase (UDP-N-acetylglucosamine:polypeptide-N-acetylglucosaminyl transferase) |
| POFUT1 | protein O-fucosyltransferase 1 |
| POFUT2 | protein O-fucosyltransferase 2 |
| POMGNT1 | protein O-linked mannose beta1,2-N-acetylglucosaminyltransferase |
| POMT1 | protein-O-mannosyltransferase 1 |
| POMT2 | protein-O-mannosyltransferase 2 |
| RFNG | RFNG O-fucosylpeptide 3-beta-N-acetylglucosaminyltransferase |
| SLC35A1 | solute carrier family 35 (CMP-sialic acid transporter), member A1 |
| SLC35A2 | solute carrier family 35 (UDP-galactose transporter), member A2 |
| SLC35A3 | solute carrier family 35 (UDP-N-acetylglucosamine (UDP-GlcNAc) transporter), member A3 |
| SLC35B1 | solute carrier family 35, member B1 |
| SLC35B2 | solute carrier family 35, member B2 |
| SLC35B3 | solute carrier family 35, member B3 |
| SLC35B4 | solute carrier family 35, member B4 |
| SLC35C1 | solute carrier family 35, member C1 |
| SLC35D1 | solute carrier family 35 (UDP-glucuronic acid/UDP-N-acetylgalactosamine dual transporter), member D1 |
| SLC35D2 | solute carrier family 35, member D2 |
| ST3GAL1 | ST3 beta-galactoside alpha-2,3-sialyltransferase 1 |
| ST3GAL2 | ST3 beta-galactoside alpha-2,3-sialyltransferase 2 |
| ST3GAL3 | ST3 beta-galactoside alpha-2,3-sialyltransferase 3 |
| ST3GAL4 | ST3 beta-galactoside alpha-2,3-sialyltransferase 4 |
| ST3GAL5 | ST3 beta-galactoside alpha-2,3-sialyltransferase 5 |
| ST3GAL6 | ST3 beta-galactoside alpha-2,3-sialyltransferase 6 |
| ST6GAL1 | ST6 beta-galactosamide alpha-2,6-sialyltranferase 1 |
| ST6GAL2 | ST6 beta-galactosamide alpha-2,6-sialyltranferase 2 |
| ST6GALNAC1 | ST6 (alpha-N-acetyl-neuraminyl-2,3-beta-galactosyl-1,3)-N-acetylgalactosaminide alpha-2,6-sialyltransferase 1 |
| ST6GALNAC2 | ST6 (alpha-N-acetyl-neuraminyl-2,3-beta-galactosyl-1,3)-N-acetylgalactosaminide alpha-2,6-sialyltransferase 2 |
| ST6GALNAC3 | ST6 (alpha-N-acetyl-neuraminyl-2,3-beta-galactosyl-1,3)-N-acetylgalactosaminide alpha-2,6-sialyltransferase 3 |
| ST6GALNAC4 | ST6 (alpha-N-acetyl-neuraminyl-2,3-beta-galactosyl-1,3)-N-acetylgalactosaminide alpha-2,6-sialyltransferase 4 |
| ST6GALNAC5 | ST6 (alpha-N-acetyl-neuraminyl-2,3-beta-galactosyl-1,3)-N-acetylgalactosaminide alpha-2,6-sialyltransferase 5 |
| ST6GALNAC6 | ST6 (alpha-N-acetyl-neuraminyl-2,3-beta-galactosyl-1,3)-N-acetylgalactosaminide alpha-2,6-sialyltransferase 6 |
| ST8SIA1 | ST8 alpha-N-acetyl-neuraminide alpha-2,8-sialyltransferase 1 |
| ST8SIA2 | ST8 alpha-N-acetyl-neuraminide alpha-2,8-sialyltransferase 2 |
| ST8SIA3 | ST8 alpha-N-acetyl-neuraminide alpha-2,8-sialyltransferase 3 |
| ST8SIA4 | ST8 alpha-N-acetyl-neuraminide alpha-2,8-sialyltransferase 4 |
| ST8SIA5 | ST8 alpha-N-acetyl-neuraminide alpha-2,8-sialyltransferase 5 |
| ST8SIA6 | ST8 alpha-N-acetyl-neuraminide alpha-2,8-sialyltransferase 6 |
| UGCG | UDP-glucose ceramide glucosyltransferase |
| UGGT1 | UDP-glucose ceramide glucosyltransferase-like 1 |
| UGGT2 | UDP-glucose ceramide glucosyltransferase-like 2 |
| UGT8 | UDP glycosyltransferase 8 |
| UST | uronyl-2-sulfotransferase |
| WBSCR17 | Williams-Beuren syndrome chromosome region 17 |
| XYLT1 | xylosyltransferase I |
| XYLT2 | xylosyltransferase II |
