# Supplementary material for: Identification of glycogene signature as a tool to predict the clinical outcome and immunotherapy response in breast cancer
Source: Front Oncol. 2022 Sep 14;12:854284. doi: 10.3389/fonc.2022.854284 (PMC9515430; doi:10.3389/fonc.2022.854284)
Supplement: Supplementary file 4 [file Table_2.docx]

**Supplementary Table 2 A total of 131 glycogenes were found to be differentially expressed between breast cancer and normal tissues**

| **Genes** | **Normal** | **Tumor** | **logFC** | **P Value** | **FDR** |
| --- | --- | --- | --- | --- | --- |
| A4GALT | 3.115337 | 2.647898 | -0.46744 | 9.99E-13 | 1.67E-12 |
| ABO | 1.159209 | 0.718539 | -0.44067 | 6.65E-38 | 2.47E-37 |
| ALG1 | 2.179315 | 2.818852 | 0.639537 | 9.56E-61 | 8.08E-60 |
| ALG10 | 0.847815 | 1.07263 | 0.224815 | 2.68E-17 | 5.18E-17 |
| ALG12 | 2.124196 | 2.023049 | -0.10115 | 0.012447 | 0.013976 |
| ALG13 | 2.921624 | 2.248397 | -0.67323 | 4.75E-22 | 1.06E-21 |
| ALG14 | 1.064027 | 1.233141 | 0.169114 | 6.47E-12 | 1.05E-11 |
| ALG2 | 3.45447 | 3.943775 | 0.489305 | 4.76E-20 | 1E-19 |
| ALG3 | 3.352522 | 3.992523 | 0.64 | 4.56E-44 | 2.07E-43 |
| ALG5 | 3.915337 | 4.016418 | 0.10108 | 0.000543 | 0.000653 |
| ALG6 | 2.001992 | 2.444135 | 0.442143 | 3.93E-46 | 1.94E-45 |
| ALG8 | 3.556125 | 4.12087 | 0.564746 | 3.37E-30 | 9.72E-30 |
| ALG9 | 2.220488 | 1.051769 | -1.16872 | 6.43E-56 | 4.51E-55 |
| B3GALNT1 | 2.10857 | 3.079236 | 0.970666 | 5.84E-59 | 4.62E-58 |
| B3GALNT2 | 1.593637 | 1.878289 | 0.284652 | 1.57E-14 | 2.78E-14 |
| B3GALT4 | 2.128876 | 2.517121 | 0.388245 | 1.56E-19 | 3.22E-19 |
| B3GALT6 | 2.702205 | 3.571619 | 0.869414 | 1.27E-52 | 7.95E-52 |
| B3GAT3 | 3.644307 | 4.139198 | 0.494892 | 2.54E-23 | 5.91E-23 |
| B3GNT3 | 1.678894 | 1.346325 | -0.33257 | 1.77E-06 | 2.36E-06 |
| B3GNT5 | 1.492206 | 0.985575 | -0.50663 | 8.87E-35 | 2.96E-34 |
| B3GNT7 | 0.905854 | 1.306787 | 0.400933 | 2.09E-11 | 3.33E-11 |
| B3GNT8 | 1.216096 | 1.649967 | 0.433871 | 3.52E-18 | 6.96E-18 |
| B4GALNT3 | 1.044115 | 2.278885 | 1.23477 | 5.37E-45 | 2.54E-44 |
| B4GALNT4 | 1.7472 | 3.044273 | 1.297074 | 2.89E-49 | 1.61E-48 |
| B4GALT1 | 5.012822 | 5.962012 | 0.94919 | 1.33E-33 | 4.27E-33 |
| B4GALT2 | 3.540382 | 4.127736 | 0.587354 | 3.27E-39 | 1.27E-38 |
| B4GALT3 | 3.581712 | 4.424298 | 0.842586 | 6.91E-70 | 8.73E-69 |
| B4GALT4 | 2.015648 | 2.276646 | 0.260998 | 3.83E-18 | 7.57E-18 |
| B4GALT6 | 1.071769 | 0.762005 | -0.30976 | 1.86E-21 | 4.09E-21 |
| B4GALT7 | 2.629312 | 2.736714 | 0.107402 | 0.003795 | 0.004381 |
| C1GALT1 | 2.122435 | 1.968058 | -0.15438 | 6.26E-07 | 8.49E-07 |
| C1GALT1C1 | 3.513248 | 4.243811 | 0.730563 | 5.32E-30 | 1.53E-29 |
| CHPF | 3.824285 | 5.313277 | 1.488992 | 5.01E-78 | 9.6E-77 |
| CHPF2 | 3.256044 | 4.289132 | 1.033088 | 5.79E-76 | 1E-74 |
| CHST1 | 1.025821 | 2.208764 | 1.182944 | 9.7E-51 | 5.71E-50 |
| CHST10 | 1.665755 | 2.285519 | 0.619764 | 5.36E-38 | 2E-37 |
| CHST11 | 1.487523 | 2.535797 | 1.048275 | 1.58E-69 | 1.95E-68 |
| CHST12 | 1.768094 | 1.222268 | -0.54583 | 2.94E-14 | 5.17E-14 |
| CHST14 | 2.891911 | 3.170997 | 0.279086 | 1.9E-22 | 4.28E-22 |
| CHST15 | 2.874563 | 3.606332 | 0.731769 | 5.16E-38 | 1.93E-37 |
| CHST2 | 1.829005 | 1.434201 | -0.3948 | 1.34E-24 | 3.23E-24 |
| CHST3 | 3.035878 | 2.087219 | -0.94866 | 2.52E-42 | 1.08E-41 |
| CHST7 | 1.741676 | 1.206613 | -0.53506 | 1.28E-40 | 5.2E-40 |
| CHST8 | 0.706713 | 1.45037 | 0.743657 | 8.74E-08 | 1.22E-07 |
| CHSY1 | 3.792341 | 4.02011 | 0.227769 | 3.96E-05 | 5E-05 |
| CHSY3 | 0.919028 | 1.226227 | 0.3072 | 3.23E-14 | 5.68E-14 |
| CSGALNACT1 | 2.189563 | 1.656088 | -0.53347 | 1.07E-32 | 3.34E-32 |
| CSGALNACT2 | 2.964495 | 3.378636 | 0.414141 | 4.35E-21 | 9.47E-21 |
| DPAGT1 | 3.301551 | 3.395915 | 0.094364 | 0.001716 | 0.002011 |
| DPM1 | 4.469101 | 5.079415 | 0.610314 | 2E-31 | 6.01E-31 |
| DPM2 | 3.507956 | 4.252011 | 0.744056 | 4.57E-71 | 6.1E-70 |
| DPM3 | 4.648877 | 4.944664 | 0.295787 | 5.03E-07 | 6.85E-07 |
| EXT1 | 3.108805 | 3.014176 | -0.09463 | 0.000213 | 0.000261 |
| EXTL2 | 2.19915 | 2.068583 | -0.13057 | 7.78E-05 | 9.7E-05 |
| FKRP | 2.142628 | 1.89057 | -0.25206 | 1.16E-14 | 2.06E-14 |
| FKTN | 1.804294 | 2.231995 | 0.427701 | 1.34E-29 | 3.79E-29 |
| FUT1 | 1.245103 | 1.418577 | 0.173474 | 0.001174 | 0.001388 |
| FUT10 | 1.358975 | 1.190906 | -0.16807 | 8.28E-12 | 1.34E-11 |
| FUT2 | 0.81609 | 1.752041 | 0.935951 | 9.39E-43 | 4.08E-42 |
| FUT8 | 2.646508 | 3.498814 | 0.852306 | 3.4E-40 | 1.36E-39 |
| GAL3ST4 | 1.886962 | 2.661604 | 0.774642 | 1.14E-44 | 5.35E-44 |
| GALNT1 | 4.057479 | 4.831809 | 0.774329 | 3.86E-25 | 9.51E-25 |
| GALNT10 | 2.952293 | 3.61069 | 0.658397 | 2.88E-26 | 7.34E-26 |
| GALNT11 | 3.267835 | 2.919237 | -0.3486 | 1.65E-38 | 6.26E-38 |
| GALNT12 | 1.371162 | 0.882698 | -0.48846 | 8.51E-33 | 2.67E-32 |
| GALNT15 | 3.963237 | 1.355211 | -2.60803 | 3.79E-87 | 1.33E-85 |
| GALNT2 | 3.311784 | 3.783382 | 0.471598 | 7.22E-29 | 2E-28 |
| GALNT5 | 0.67676 | 1.529221 | 0.852461 | 1.93E-29 | 5.43E-29 |
| GALNT6 | 2.746123 | 4.462617 | 1.716494 | 1.66E-41 | 6.92E-41 |
| GALNT7 | 2.757706 | 3.943358 | 1.185653 | 1.67E-40 | 6.75E-40 |
| GBGT1 | 1.718531 | 1.429029 | -0.2895 | 5.12E-16 | 9.52E-16 |
| GCNT1 | 1.747713 | 2.389998 | 0.642285 | 1.58E-16 | 2.98E-16 |
| GCNT4 | 0.933764 | 0.688798 | -0.24497 | 2.34E-15 | 4.27E-15 |
| HAS2 | 1.00133 | 1.146664 | 0.145334 | 7.83E-08 | 1.1E-07 |
| HAS3 | 2.629044 | 0.899643 | -1.7294 | 1.28E-62 | 1.17E-61 |
| HS2ST1 | 2.323659 | 2.782124 | 0.458465 | 7.07E-15 | 1.27E-14 |
| HS3ST1 | 0.767372 | 0.729375 | -0.038 | 0.015914 | 0.017759 |
| HS3ST2 | 0.899437 | 0.797021 | -0.10242 | 0.031262 | 0.034349 |
| HS3ST3A1 | 0.204607 | 0.555075 | 0.350467 | 4.57E-46 | 2.25E-45 |
| HS3ST3B1 | 0.665692 | 0.959184 | 0.293491 | 7E-17 | 1.33E-16 |
| HS6ST1 | 3.321076 | 4.009014 | 0.687938 | 3.76E-42 | 1.61E-41 |
| HS6ST3 | 0.218718 | 1.026024 | 0.807306 | 2.38E-21 | 5.21E-21 |
| LFNG | 3.071594 | 3.805117 | 0.733524 | 2.18E-16 | 4.09E-16 |
| MFNG | 2.800719 | 2.070956 | -0.72976 | 4.21E-35 | 1.42E-34 |
| MGAT1 | 5.171044 | 4.672168 | -0.49888 | 1.98E-29 | 5.56E-29 |
| MGAT2 | 1.242207 | 0.891058 | -0.35115 | 0.000609 | 0.00073 |
| MGAT3 | 1.673132 | 0.91352 | -0.75961 | 8.61E-42 | 3.63E-41 |
| MGAT4A | 2.578503 | 3.351982 | 0.773479 | 7.13E-32 | 2.18E-31 |
| MGAT4B | 4.092336 | 4.247658 | 0.155323 | 6.39E-05 | 8E-05 |
| MGAT5 | 2.703626 | 3.361098 | 0.657472 | 7.32E-13 | 1.23E-12 |
| NDST2 | 1.768184 | 0.700341 | -1.06784 | 3.11E-08 | 4.43E-08 |
| OGT | 5.062651 | 4.137491 | -0.92516 | 4.27E-45 | 2.03E-44 |
| POFUT1 | 3.473242 | 4.146002 | 0.67276 | 5.2E-26 | 1.31E-25 |
| POFUT2 | 2.98025 | 2.732414 | -0.24784 | 1.57E-07 | 2.18E-07 |
| POMGNT1 | 3.746206 | 3.893194 | 0.146988 | 1.9E-06 | 2.53E-06 |
| POMT1 | 3.078626 | 2.76862 | -0.31001 | 9.95E-11 | 1.55E-10 |
| POMT2 | 2.272314 | 1.907236 | -0.36508 | 8.37E-13 | 1.4E-12 |
| RFNG | 3.626769 | 3.374749 | -0.25202 | 7.05E-07 | 9.54E-07 |
| SLC35A1 | 3.304899 | 3.399319 | 0.09442 | 0.018821 | 0.02094 |
| SLC35A2 | 2.954042 | 4.205061 | 1.251019 | 1.7E-99 | 3.39E-97 |
| SLC35A3 | 2.630447 | 3.370376 | 0.739929 | 4.28E-35 | 1.44E-34 |
| SLC35B1 | 3.279119 | 3.427916 | 0.148798 | 0.000929 | 0.001103 |
| SLC35B2 | 4.207118 | 5.086741 | 0.879624 | 6.51E-45 | 3.07E-44 |
| SLC35B3 | 3.259856 | 3.944985 | 0.685129 | 1.29E-29 | 3.66E-29 |
| SLC35B4 | 1.719845 | 1.811654 | 0.09181 | 0.018805 | 0.020925 |
| SLC35C1 | 2.794574 | 2.981517 | 0.186944 | 0.003604 | 0.004164 |
| SLC35D1 | 1.985169 | 2.690258 | 0.705089 | 2.84E-34 | 9.31E-34 |
| SLC35D2 | 3.271536 | 3.33849 | 0.066954 | 0.009673 | 0.01093 |
| ST3GAL1 | 2.900435 | 3.794409 | 0.893974 | 4.23E-36 | 1.47E-35 |
| ST3GAL2 | 1.881489 | 1.777592 | -0.1039 | 0.001049 | 0.001242 |
| ST3GAL3 | 2.408621 | 1.760233 | -0.64839 | 1.38E-58 | 1.08E-57 |
| ST3GAL4 | 2.669317 | 3.105801 | 0.436484 | 5.14E-14 | 8.95E-14 |
| ST3GAL5 | 2.459813 | 1.835458 | -0.62436 | 1.63E-28 | 4.46E-28 |
| ST3GAL6 | 1.761424 | 1.137865 | -0.62356 | 1.47E-50 | 8.59E-50 |
| ST6GAL1 | 3.30475 | 3.193354 | -0.1114 | 0.037587 | 0.041097 |
| ST6GAL2 | 0.513104 | 1.252949 | 0.739844 | 1.13E-40 | 4.62E-40 |
| ST6GALNAC1 | 0.894033 | 0.459437 | -0.4346 | 3.09E-49 | 1.72E-48 |
| ST6GALNAC2 | 2.6186 | 2.888761 | 0.270161 | 2.19E-05 | 2.79E-05 |
| ST6GALNAC3 | 1.282206 | 0.458026 | -0.82418 | 4.74E-74 | 7.34E-73 |
| ST6GALNAC4 | 2.840432 | 3.070675 | 0.230243 | 0.000256 | 0.000312 |
| ST6GALNAC5 | 1.324884 | 1.683574 | 0.35869 | 0.006655 | 0.007581 |
| ST6GALNAC6 | 4.594009 | 3.380782 | -1.21323 | 5.43E-74 | 8.37E-73 |
| ST8SIA1 | 1.272692 | 0.547886 | -0.72481 | 3.56E-53 | 2.27E-52 |
| ST8SIA4 | 1.113237 | 1.560256 | 0.447019 | 2.21E-27 | 5.85E-27 |
| ST8SIA6 | 1.465984 | 2.134823 | 0.668839 | 1E-09 | 1.51E-09 |
| UGCG | 3.70729 | 4.802066 | 1.094776 | 1.52E-30 | 4.45E-30 |
| UGGT1 | 2.704316 | 3.525401 | 0.821085 | 5.07E-41 | 2.08E-40 |
| UGGT2 | 2.363341 | 2.04041 | -0.32293 | 2.07E-17 | 4.02E-17 |
| UGT8 | 1.079693 | 0.787935 | -0.29176 | 2.67E-17 | 5.15E-17 |
| UST | 2.098767 | 1.477947 | -0.62082 | 8.18E-30 | 2.33E-29 |
| XYLT2 | 2.692782 | 3.233897 | 0.541115 | 7.78E-34 | 2.52E-33 |
| gene | conMean | treatMean | logFC | pValue | fdr |
| A4GALT | 3.115337 | 2.647898 | -0.46744 | 9.99E-13 | 1.67E-12 |
| ABO | 1.159209 | 0.718539 | -0.44067 | 6.65E-38 | 2.47E-37 |
| ALG1 | 2.179315 | 2.818852 | 0.639537 | 9.56E-61 | 8.08E-60 |
| ALG10 | 0.847815 | 1.07263 | 0.224815 | 2.68E-17 | 5.18E-17 |
| ALG12 | 2.124196 | 2.023049 | -0.10115 | 0.012447 | 0.013976 |
| ALG13 | 2.921624 | 2.248397 | -0.67323 | 4.75E-22 | 1.06E-21 |
| ALG14 | 1.064027 | 1.233141 | 0.169114 | 6.47E-12 | 1.05E-11 |
| ALG2 | 3.45447 | 3.943775 | 0.489305 | 4.76E-20 | 1E-19 |
| ALG3 | 3.352522 | 3.992523 | 0.64 | 4.56E-44 | 2.07E-43 |
| ALG5 | 3.915337 | 4.016418 | 0.10108 | 0.000543 | 0.000653 |
| ALG6 | 2.001992 | 2.444135 | 0.442143 | 3.93E-46 | 1.94E-45 |
| ALG8 | 3.556125 | 4.12087 | 0.564746 | 3.37E-30 | 9.72E-30 |
| ALG9 | 2.220488 | 1.051769 | -1.16872 | 6.43E-56 | 4.51E-55 |
| B3GALNT1 | 2.10857 | 3.079236 | 0.970666 | 5.84E-59 | 4.62E-58 |
| B3GALNT2 | 1.593637 | 1.878289 | 0.284652 | 1.57E-14 | 2.78E-14 |
| B3GALT4 | 2.128876 | 2.517121 | 0.388245 | 1.56E-19 | 3.22E-19 |
| B3GALT6 | 2.702205 | 3.571619 | 0.869414 | 1.27E-52 | 7.95E-52 |
| B3GAT3 | 3.644307 | 4.139198 | 0.494892 | 2.54E-23 | 5.91E-23 |
| B3GNT3 | 1.678894 | 1.346325 | -0.33257 | 1.77E-06 | 2.36E-06 |

FC, fold change; FDR, false discovery rate.
